# Geographical, racial and socio-economic variation in life expectancy in the US and their impact on cancer relative survival

**DOI:** 10.1371/journal.pone.0201034

**Published:** 2018-07-25

**Authors:** Angela B. Mariotto, Zhaohui Zou, Christopher J. Johnson, Steve Scoppa, Hannah K. Weir, Bin Huang

**Affiliations:** 1 Division of Cancer Control and Population Sciences, National Cancer Institute, Bethesda, Maryland, United States of America; 2 Information Management Services, Calverton, Maryland, United States of America; 3 Cancer Data Registry of Idaho, Boise, Idaho, United States of America; 4 Division of Cancer Prevention and Control, Centers for Disease Control and Prevention, Atlanta, Georgia, United States of America; 5 College of Public Health, University of Kentucky, Lexington, Kentucky, United States of America; Public Library of Science, UNITED KINGDOM

## Abstract

**Purpose:**

Despite gains in life expectancy between 1992 to 2012, large disparities in life expectancy continue to exist in the United States between subgroups of the population. This study aimed to develop detailed life tables (LT), accounting for mortality differences by race, geography, and socio-economic status (SES), to more accurately measure relative cancer survival and life expectancy patterns in the United States.

**Methods:**

We estimated an extensive set of County SES-LT by fitting Poisson regression models to deaths and population counts for U.S. counties by age, year, gender, race, ethnicity and county-level SES index. We reported life expectancy patterns and evaluated the impact of the County SES-LT on relative survival using data from the Surveillance Epidemiology and End Results (SEER) Program cancer registries.

**Results:**

Between 1992 and 2012, the largest increase in life expectancy was among black men (6.8 years), however there were still large geographical differences. Life expectancy was highest for Asian or Pacific Islanders (API), and lowest for American Indians and Alaskan Natives (AIAN). In 2010, life expectancies by state ranged from 73 to 82 years for white males, 78 to 86 years for white females, 66 to 75 for black males, and 75 to 81 for black females. Comparisons of relative survival using National LT and the new County SES-LT showed that relative survival using County SES-LT improved relative survival estimates for some demographic groups, particularly in low and high SES areas, among Hispanics and AIAN, and among older male cancer patients. Relative survival using County SES-LT was 7.3% and 6.7% survival points closer to cause-specific survival compared to the National LT relative survival for AIAN and Hispanic cancer patients diagnosed between ages 75 and 84 years, respectively. Importantly, the County SES-LT relative survival estimates were higher in lower SES areas and lower in higher SES areas, reducing differences in relative survival comparisons.

**Conclusion:**

The use of these new socio-economic life tables (County SES-LT) can provide more accurate estimates of relative survival, improve comparisons of relative survival among registries, better illustrate disparities and cancer control efforts, and should be used as default for cancer relative survival using U.S. data.

## Introduction

Life tables (LT) are an important tool for calculating life expectancies [1–3] and also for the calculation of relative survival[4–6]. Relative survival is the standard method for reporting of survival from cancer registry data, as it does not rely on cause of death information from death certificates which may be missing or misclassified [7]. Relative survival is calculated as the ratio of cancer patients observed all-cause survival to the “expected” survival these patients would have in the absence of a cancer diagnosis. The expected survival, also denoted as background mortality, is calculated from population LT by matching each cancer patient in the study to their respective LT by characteristics that may affect their chances of dying from other causes.

The default LT currently used to report relative survival from the Surveillance Epidemiology and End Results (SEER) Program data [8] are national LT by sex, individual ages 0–99, race (whites, blacks and other races combined) and individual calendar years 1970–2011 [9]. These LT, herein referred to as US-LT, were constructed from decennial and annual LT from the National Center of Health Statistics (NCHS) for all races [10], whites and blacks and from NCHS mortality data for other races. Accuracy of relative survival crucially depends on how good LT represent the background mortality for the cohort of cancer patients. A recent study [11] showed that LT by state for the white and black populations captured some of the geographical variability in non-cancer cause mortality and improved relative survival calculations for younger ages but were biased for older ages. Additionally, there is evidence of variations in health status and mortality in the U.S. by geography, and by SES within the same race group[12]. Thus, national LT that do not account for variations in mortality by geography, by SES, or by race may lead to biased relative survival estimates [6, 11]. As more cancer registries in the U.S. are beginning to report relative survival it is important to have LT that represent each registry background mortality for their population to have more fair comparisons of relative survival[13]. National LT representing the average US mortality may overestimate differences in relative survival between groups of cancer patients with different mortality patterns compared to the national average. Average national LT overestimate the expected survival in more deprived areas, and underestimate expected survival in less deprived areas. Since expected survival is in the denominator for relative survival calculations, the consequence is underestimated (lower) and overestimated (higher) relative survival in more versus less deprived areas, respectively, and an increase in differences.

The goal of this study is to present a more comprehensive set of LT that more accurately represent the varying mortality patterns in different populations in the U.S. with respect to geography (county of residence), SES at the county level and individual characteristics race and ethnicity, calendar year of death, and sex. To do this, we used U.S. mortality data from 1992 through 2012 linked to SES indicators at the county level. We estimated LT by sex, calendar year (1992–2012), race (whites, blacks, Asian or Pacific Islanders (API), American Indians and Alaskan Natives (AIAN), and Hispanic-origin), state, and county-level SES as data allowed. We summarized LT by calculating life expectancies and describing patterns by state and calendar year trends by sex and race. We investigated the impact of the County SES-LT on expected and relative survival by comparing with estimates using the US-LT.

Relative survival is the preferred method to report and compare survival between different registries and countries [14] because cause of death is often unavailable, misclassified, or subject to variability on its accurate determination[7]. When cause of death is available and accurate, cause-specific survival is an alternative measure to quantify survival associated with a cancer diagnosis. An algorithm that more accurately attributes a cause of death to cancer [15] has made it possible for SEER to calculate cause-specific survival. Comparisons of relative survival and cause-specific survival are challenging because both measures are subject to bias[4].

To minimize bias, we compared survival for cancer patients diagnosed with any cancer. Because this is a broad group of cancer patients they are more likely to have similar background mortality as the general population and be less affected by bias due to cause of death determination. We hypothesized that the relative survival closer to cause-specific survival reflects the more accurate life table and relative survival.

## Data and methods

### County level mortality data

We used counts of deaths from the NCHS and populations from the U.S. Census Bureau available through SEER*Stat software [16] by county, single year age at death (30 to 84 years), race, sex, and calendar year 1992–2013. County is the smallest geographical area for which mortality data are available. We created mutually exclusive race and ethnicity groups, herein referred as race groups: Non-Hispanic (NH) White, NH Black, NH AIAN, NH API, and Hispanics (hereafter we exclude the NH prefix when referencing the race groups). Hispanic ethnicity includes all race categories. Because of misclassification errors of AIAN race in death certificates [17], we restricted the AIAN data to mortality rates from Contract Health Service Delivery Area (CHSDA) counties. The CHSDA counties in general contain federally recognized tribal land or are adjacent to tribal land and have health services for the AIAN populations supported by the Indian Health Service. Restricting analyses to CHSDA areas reduces AIAN misclassification on death certificates [17] and produces more accurate estimates of mortality for the AIAN populations.

### County level SES index

NCHS mortality data do not contain individual level SES for deceased individuals. Therefore, we used an ecologically-defined SES index linked to mortality data at the county level. We obtained SES characteristics at the county level from the U.S. 1990 and 2000 decennial censuses and from the American Community Survey (ACS) 5-year estimates for 2005–2009, 2006–2010 and 2007–2011, used to represent the average SES in years 2007, 2008 and 2009 respectively. The SES index was previously developed and validated [18]. It used factor analyses to construct a single SES index that included poverty, unemployment, occupation, income, education, and housing characteristic [18]. We used extrapolation methods to estimate SES for the missing years, for example, the SES index for years 1995 through 1999 were estimated by extrapolating the 1990 and 2000 SES indexes. We ranked US counties from lowest to highest based on the SES index, and created equal quintiles based on combined population size: Q1 (lowest SES) were counties with 20% of the US population with the lowest SES index and Q5 (highest SES) were counties with 20% of the population with the highest SES index.

### LT modeling and life expectancy calculation

We fit Poisson regression models to the log of mortality rates to estimate LT separately for men and women and each race. We used 3-year grouped mortality rates (1991–1993 for 1992, …, 2012–2014 for 2013) to provide smoothed and more stable estimates. Age and calendar year were modeled as spline functions to capture non-linear effects. Because of small numbers of deaths for younger ages and populations not being available for single ages at death for ages 85 and older, we restricted the Poisson regression model to the log of mortality rates for ages between 30 and 84 years.

Let *D*(*i,age,year*|*s,r,A*) and *P*(*i,age,year*|*s,r,A*) be the number of race r and sex s deaths and population at county *i* in area *A* by age (age = 30, …., 84) and year where year represents the midpoint of the 3 calendar years. The model assumes that the numbers of deaths follow a Poisson distribution with mean that is the product of the population and the mortality rate in a given cell, *D*(*i,age,year*|*s,r,A*)~Poisson[*P*(*i,age,year*|*s,r,A*)*λ*(*i,age,year*|*s,r,A*)]. The models varied by geographic area (state, region, and national) and the inclusion or not of the SES index as a covariate depending on sufficient numbers of deaths and population counts for each race. SES was included either as 5 level quintiles or 2 level grouped into low SES Q1-Q3 vs. high SES Q4-Q5. This grouping was used to maximize SES differences and ensure sufficient number of deaths and populations in the two groups. For each area (state, region or national), race and sex, the log of mortality rates was modeled as a spline function of continuous age and calendar year. The models are:

1. including SES quintiles as a 5-level covariate
  $$\ln\left[\lambda (i,age,year|sex,race)\right]=f(age)+g(year)+\sum_{j=2}^{5}\beta_{j}{\left(SES\;Q\right)}_{i}+\sum_{j=2}^{5}\gamma_{j}{\left(SES\;Q\right)}_{i}*age$$
  where county *i* belongs to the respective area, *f* and *g* are restricted cubic spline functions.
2. including SES as a 2-level covariate
  $$\ln\left[\lambda (i,age,year|sex,black)\right]=f(age)+g(year)+\beta {\left(HighSES\right)}_{i}+\gamma {\left(HighSES\right)}_{i}*age.$$
3. without SES, ln[*λ*(*i,age,year*|*sex,black*)] = *f*(*age*)+*g*(*year*).

In all models the nodes for the spline function of age are fixed at 32, 40, 55, 70, 77, 82 and for year are 1992, 1997, 2002, 2007, and 2012. We used SAS PROC GENMOD to fit the Poisson models.

To estimate LT for ages 0 to 34 ears and 85 to 99 years we used an adjustment based on the race, sex and year-specific NCHS decennial US-LT [19]. The NCHS decennial LT have improved estimates for these age groups because they include extra data on births and better information on age at death for the very old from linkages to Medicare data [19]. The idea of the adjustment is to keep the level of the modeled LT as estimated for ages 35 and 84 and to use the form of the mortality rate by age from the US-LT to extrapolate beyond those ages. Let *λ_US_*(*age,year*|*sex,race*) be the probability of dying between age and age+1 from decennial US-LT and $\hat{\lambda }(age,year|sex,race)$ the state, region or national estimated LT for the respective race and sex. For ages *a*<35 and *a*>84 we adjusted the estimates as below,

$$\hat{\lambda }(a,year|sex,race)=\hat{\lambda }(35,year|sex,race)\frac{\lambda_{US}(a,year|sex,race)}{\lambda_{US}(35,year|sex,race)},\;a<35$$

and

$$\hat{\lambda }(a,year|sex,race)=\hat{\lambda }(84,year|sex,race)\frac{\lambda_{US}(a,year|sex,race)}{\lambda_{US}(84,year|sex,race)},\;a>84.$$

For whites and all races combined, we fit the models for each state with county SES included as a covariate with 5 levels. The models varied for the other race groups depending on sufficient deaths and populations counts at each state. The models were: state and no SES, state and 2-level SES. For states with small populations for the respective race, LT were estimated using their respective regions and 5-level SES for Blacks and Hispanics and national with 2-level SES for API and AIAN. The models used for each state and race combination are shown in S1 Table.

To compare the County SES-LT and summarize the effects of year, sex, race, state, and county-level SES we calculated life expectancy up to age 99 using standard LT methods [20].

### Comparisons of expected and relative survival

SEER collects clinical, demographic, and vital status information on all cancer cases diagnosed in defined geographic areas. Data included in this report are from SEER-18 registries (2000–2012) (November 2015 Submission) obtained using the SEER*Stat Version 8.3.2 software [21] covering approximately 30% of the US population. Relative survival is defined as the ratio of overall survival (all causes of death) by the expected survival in a comparable group of cancer free individuals and represents the excess mortality from a cancer diagnosis. Currently expected survival is estimated from US-LT matched to the group of cancer patients by age, sex, race, and calendar year. We used the Ederer II method to calculate expected survival [22, 23]. The new County SES-LT were incorporated into SEER*Stat software. Relative survival calculations match individuals in the survival cohort to the County SES-LT by age, sex, race, calendar year and county of residence at the time of cancer diagnosis. SES is accounted for through the county of residence.

We selected patients diagnosed between 2000 and 2012 in the 18 SEER registries with any cancer, to calculate 5-year and 10-year expected survival, relative survival using the US-LT [9], relative survival using the new County SES-LT and cause-specific survival. In this paper, we report 10-year survival because it maximizes differences. We censored individuals when they reached age 99. We excluded patients diagnosed by autopsy or death certificate and those with no follow-up information (zero survival time).

Cause-specific survival uses cancer death as the endpoint and censors people dying of other causes, at the end of the study date or at attained age of 99 years, whichever comes first. Because of inherent ambiguities in determining the underlying cause of death (for example, a metastatic site reported as the cause of death rather than the original cancer site [7], SEER developed a cause-specific death classification algorithm [15, 24] to better code deaths related to the specific cancer. This algorithm uses causes of deaths that are likely to be related to the cancer or because of a cancer diagnosis. In the comparisons between relative and cause-specific survival we only included people with one primary malignant cancer, as cause of death is more likely to be misclassified for people diagnosed with multiple tumors. We did not report survival statistics when the number of patients at diagnosis were less than 50.

## Results

Table 1 displays 2010 state population counts for each race and the percent of the population in each SES quintile. Note that DC does not have county subdivisions, Hawaii and Alaska counties are collapsed over state, and some states contain counties with 4 or fewer quintiles.

**Table 1:** Race state population estimates in 2010 and percent of population (all races combined) in each SES quintile.

	Population (in thousands) 2010						Percent population in quintiles (%)				
	Total	NH Whites	NH Blacks	NH API	NH AIAN	Hispanic	Q1-Low	Q2	Q3	Q4	Q5-High
**Alabama**	4,757	3,243	1,267	60	7	180	48	34	4	10	4
**Alaska**	714	480	29	50	115	40	0	0	0	0	100
**Arizona**	6,412	3,766	271	200	270	1,905	11	23	66	0	0
**Arkansas**	2,893	2,207	461	-	0	181	69	17	11	4	0
**California**	36,902	15,482	2,366	5,210	113	13,731	4	10	39	17	30
**Colorado**	4,963	3,591	217	159	6	990	8	15	0	67	9
**Connecticut**	3,579	2,583	359	145	9	483	0	0	0	52	48
**Delaware**	893	596	196	-	0	70	0	0	0	40	60
**District of Columbia**	601	215	309	-	0	54	0	0	0	100	0
**Florida**	18,773	11,068	2,965	512	11	4,218	25	34	34	7	1
**Georgia**	9,660	5,500	2,989	-	0	829	35	11	14	34	7
**Hawaii**	1,358	299	30	-	0	115	0	0	0	0	100
**Idaho**	1,556	1,337	13	24	12	170	29	26	18	26	1
**Illinois**	12,753	8,264	1,893	-	0	1,973	6	12	50	10	21
**Indiana**	6,466	5,344	624	114	2	382	29	39	12	13	7
**Iowa**	3,040	2,726	103	59	4	149	9	27	23	37	4
**Kansas**	2,824	2,268	184	75	5	291	19	30	23	10	19
**Kentucky**	4,337	3,794	357	-	0	130	47	38	6	5	4
**Louisiana**	4,514	2,772	1,472	77	4	189	51	33	3	12	0
**Maine**	1,322	1,267	19	15	5	17	23	33	6	39	0
**Maryland**	5,752	3,218	1,731	-	0	456	12	1	2	6	79
**Massachusetts**	6,545	5,095	447	375	8	621	0	7	13	12	68
**Michigan**	9,836	7,660	1,449	261	38	428	42	14	10	19	14
**Minnesota**	5,271	4,464	301	230	36	240	1	21	16	32	31
**Mississippi**	2,962	1,738	1,108	29	8	80	72	8	7	13	0
**Missouri**	5,957	4,914	723	-	0	206	37	20	7	27	8
**Montana**	979	882	6	8	55	28	22	43	16	17	1
**Nebraska**	1,825	1,517	92	36	13	167	10	19	26	34	11
**Nevada**	2,703	1,501	231	226	27	719	2	2	74	22	0
**New Hampshire**	1,312	1,228	17	-	0	36	3	0	0	33	64
**New Jersey**	8,745	5,284	1,174	-	0	1,525	0	0	7	40	53
**New Mexico**	2,052	852	42	31	177	950	40	46	0	13	1
**New York**	19,212	11,462	2,924	1,512	23	3,291	9	23	11	29	29
**North Carolina**	9,424	6,319	2,089	230	15	771	33	33	8	14	12
**North Dakota**	665	605	10	8	29	13	6	24	29	37	3
**Ohio**	11,501	9,464	1,476	-	0	346	23	28	31	12	6
**Oklahoma**	3,759	2,689	307	75	355	333	36	46	2	16	0
**Oregon**	3,834	3,077	83	172	51	452	28	19	26	18	10
**Pennsylvania**	12,664	10,191	1,399	0	0	700	21	17	17	26	20
**Rhode Island**	1,044	819	64	33	1	127	0	60	0	16	25
**South Carolina**	4,636	3,005	1,309	67	18	237	47	15	27	11	0
**South Dakota**	806	699	13	9	64	21	18	24	24	27	7
**Tennessee**	6,328	4,863	1,083	-	0	281	38	44	5	9	3
**Texas**	24,979	11,619	2,983	1,035	4	9,338	22	28	18	19	12
**Utah**	2,752	2,262	34	89	18	349	2	8	6	70	14
**Vermont**	623	597	8	-	0	9	5	21	12	37	25
**Virginia**	7,979	5,290	1,589	-	0	616	18	12	6	10	54
**Washington**	6,735	5,020	285	569	103	758	9	9	9	31	43
**West Virginia**	1,850	1,744	70	-	0	22	69	19	6	6	0
**Wisconsin**	5,664	4,781	378	139	37	329	21	15	17	27	21
**Wyoming**	558	490	6	5	9	48	3	7	11	55	23

**Fig 1:**
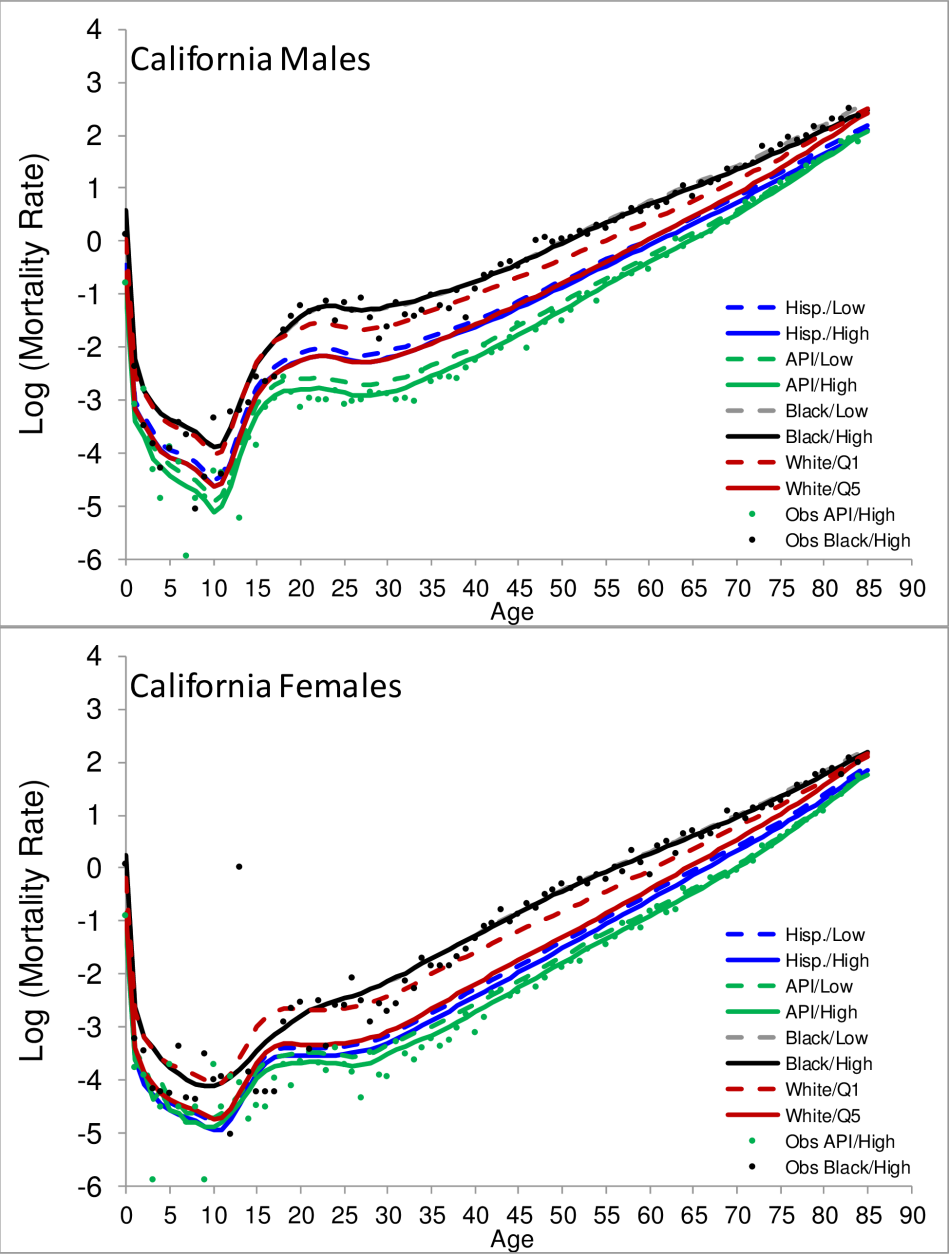
**Estimated and selected observed 2010 log mortality rates for males (A) and females (B) residing in the state of California by race and socio-economic status (SES). Observed log mortality rates are displayed for blacks and API in the high SES group (Q4-Q5).** White = Non-Hispanic (NH) White, Black = NH Black, API = NH Asian or Pacific Islander, AIAN = NH American Indian and American Natives, and Hisp. = Hispanics. For whites only, LT for the lowest (Q1) and highest (Q5) SES quintiles are shown. For blacks, Hispanics, and API we only estimated LT by 2 level SES: Low = Q1-Q3 SES and High = Q4-Q5 SES. Observed log mortality rates for blacks and for API’s are for the high SES group.

Fig 1 displays an example of the estimated County SES-LT in terms of log mortality for males and females by race in the state of California in 2010. The figure also displays the fit of observed log mortality rates for blacks and API in the high SES group (Q4-Q5). The patterns observed in California were in general similar to other states and show that: mortality is lower for API followed by Hispanics, whites, and blacks. Although not shown, AIAN have slightly lower mortality compared to blacks and there is a good fit of the County SES-LT to data. Figures for other states will be available in a website or by request.

### Trends in life expectancies by race and SES

**Fig 2:**
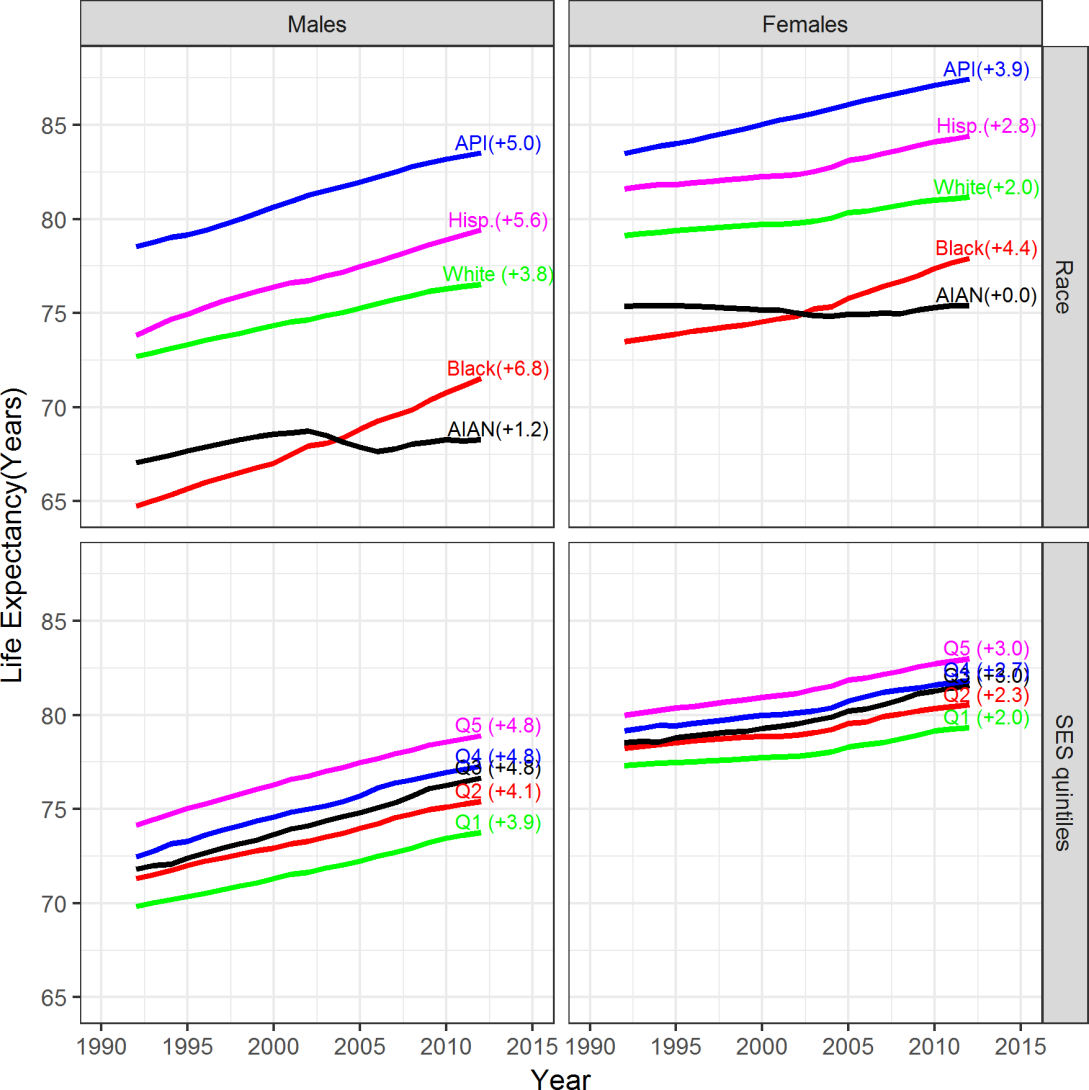
Trends in life expectancy from 1992 through 2012 for males and females by race and SES quintiles of their county of residency. Life expectancies are from birth to age 99 and the numbers in parentheses represent gains in life expectancy (in years) from 1992 to 2012.

Between 1992 and 2012, male life expectancy increased more rapidly than female life expectancy in all races (Fig 2). Black men experienced the highest gains in life expectancy, 6.8 years, followed by Hispanic men (5.6 years), API men (5.0 years) and white men (3.8 years). Life expectancies among black, API, Hispanic and white women increased 4.4 years, 3.9 years, 2.8 years, and 2.0 years respectively. AIAN men and women experienced the smallest gains in life expectancy, 1.2 and 0 years, respectively. Gains in life expectancy were slightly higher for people living in counties with higher SES (Fig 2).

### Life expectancies by state

**Fig 3:**
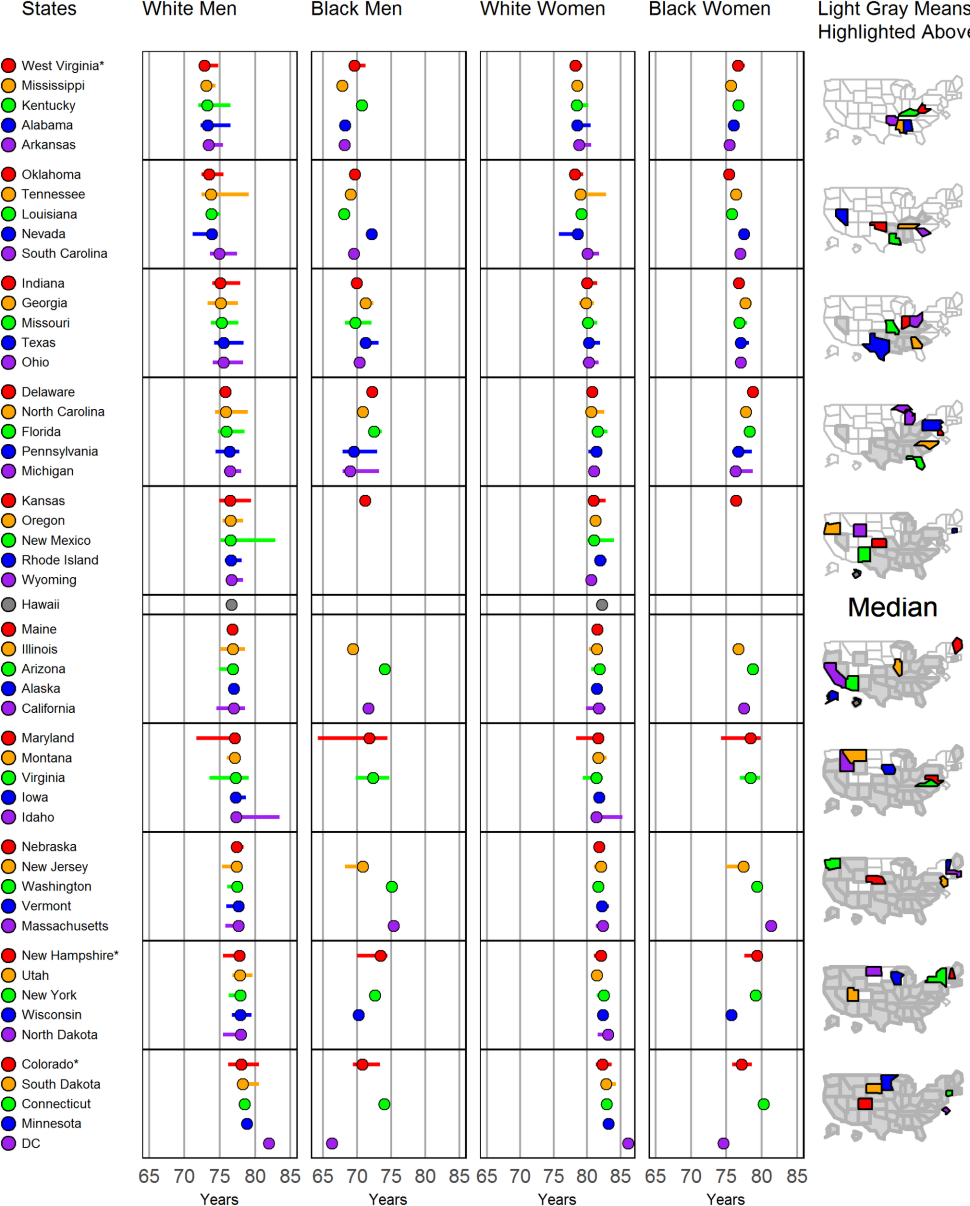
Life expectancy from birth to age 99 in 2010 by state and sex for whites and blacks, ordered by life expectancy for white males. The left and right end of the bars representing life expectancies estimates of patients living in counties with lowest and highest SES quintiles, respectively. The dots represent average life expectancy in the respective state, race and sex combination.

Fig 3 displays linked (micro-)maps to facilitate geographical visualization of clusters of life expectancies by state. The dots represent the 2010 average life expectancy (in years) by state for white males, black males, white females, and black females. The horizontal bars represent the range of life expectancies between the lowest and highest SES quintile, the left and right end of the bar being the life expectancies for the lowest and highest SES quintile, respectively. The figure orders states by white male life expectancy from lowest (West Virginia, 73 years) to highest (DC, 82 years). The ordered states are color-coded in groups of 5 (panel 1), and the colors provide a link between the life expectancy estimates for the different race and sex (panels 2–5) and the maps (panel 6). Each map uses color to highlight states in that particular group. For example, the first map colors the states with the 5 lowest white male life expectancies. As we go down the maps, the next states with lower expectancy are colored and all the previous colored states (which have lower life expectancies) are displayed in grey. In the second map West Virginia, Mississippi, Kentucky, Alabama, and Arkansas are grey and Oklahoma, Tennessee, Louisiana, Nevada and South Carolina are colored. The maps show that south-eastern states have the lowest life expectancy and north-western states have the highest. There are significant differences in life expectancy by state, ranging from 73 to 82 years for white males, 78 to 86 years for white females, 66 to 75 for black males, and 75 to 81 for black females. States with wide bars show large disparities in life expectancy within the state, e.g. Idaho, Maryland, New Mexico, Tennessee and Virginia. Washington DC has the highest life expectancy for whites and the lowest for blacks.

### Impact of LT on expected survival and relative survival

**Fig 4:**
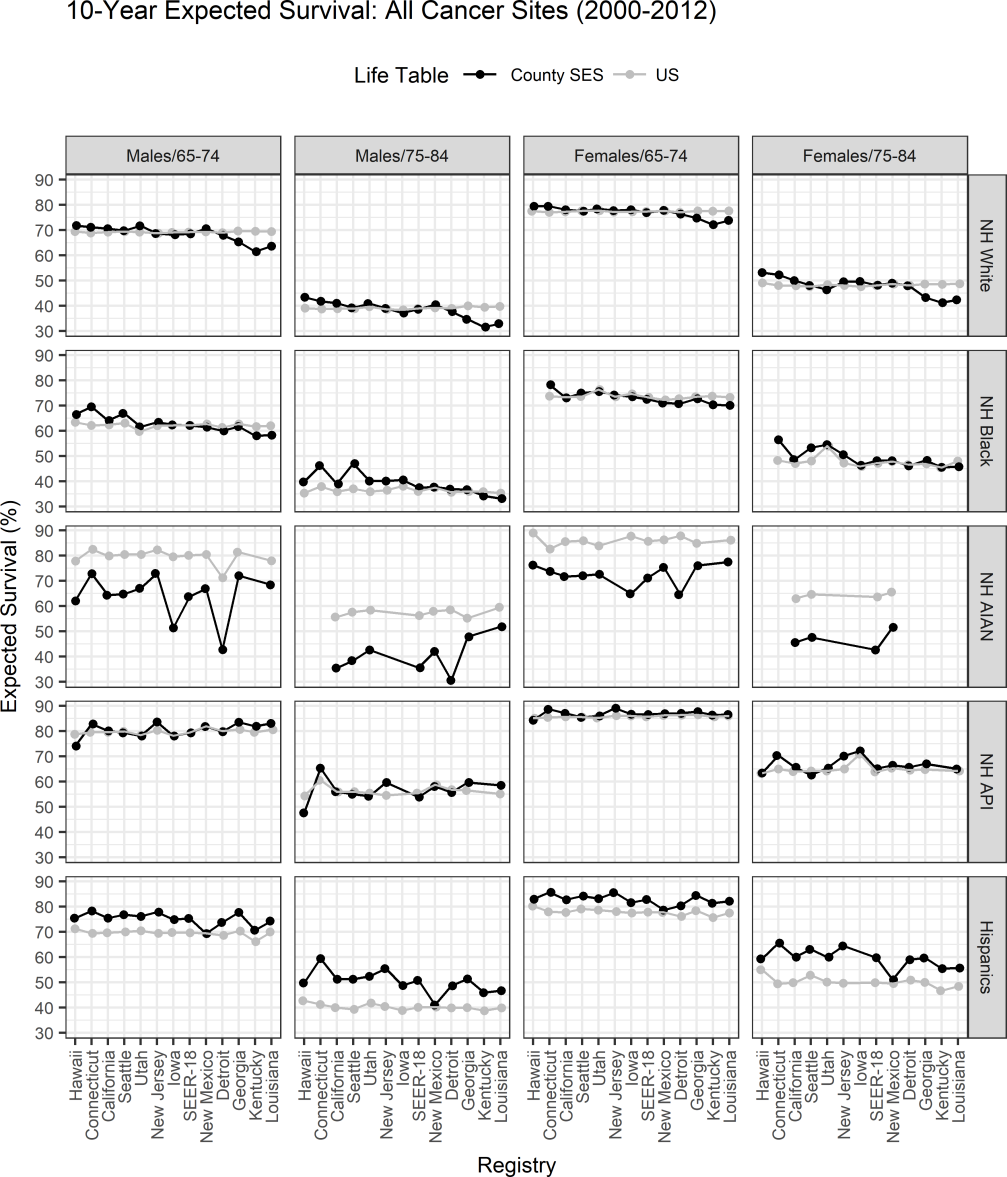
10-year expected survival for cancer patients diagnosed between 2000 and 2012 with any type of cancer by sex, race, age and geographic area calculated using the County SES-LT (black) versus the US-LT (gray). Registries are grouped from the highest SES (left of SEER-18) to the lowest SES (right of SEER-18).

Fig 4 displays 10-year expected survival for men and women diagnosed with any cancer by age and race using the County SES vs. the US-LT. Numbers can be interpreted as the percent surviving other causes of death 10 years after diagnosis estimated from each LT. Registries are ordered from higher SES to lower SES. In general, differences between expected survival from national and County SES-LT were small for whites, blacks and API’s and larger for the AIAN and Hispanics cancer patient’s populations. However, for whites and to a lesser extent for blacks there is consistent pattern between low vs. high SES areas. While the US-LT produced mostly flat expected survival the expected survival from the County SES life tables has a gradient with expected survival being higher in high SES areas (Hawaii, Connecticut, California, Seattle, Utah) and lower in low SES areas (Detroit, Georgia, Kentucky and Louisiana). The effect is higher for white, males and older ages 75–84. We are not able to observe this gradient for the County SES expected survival for AIAN, API and Hispanics because we were not able to estimate state specific LT for many states. The US and County SES expected survival were very similar for APIs with the largest differences occurring for males in Hawaii, and state for which there is state specific County SES-LT. The largest difference between US and County SES expected survival was observed for AIAN cancer population. The County SES expected survival is much lower, often 20% points lower than US expected survival, as the US-LT was available for other races, grouping AIAN with API mortality data. Because the API population is larger compared to the AIAN population, the US other race LT better reflect API’s than AIANs. The Hispanic expected survival is higher than the expected survival from US-LT, except for New Mexico.

**Fig 5:**
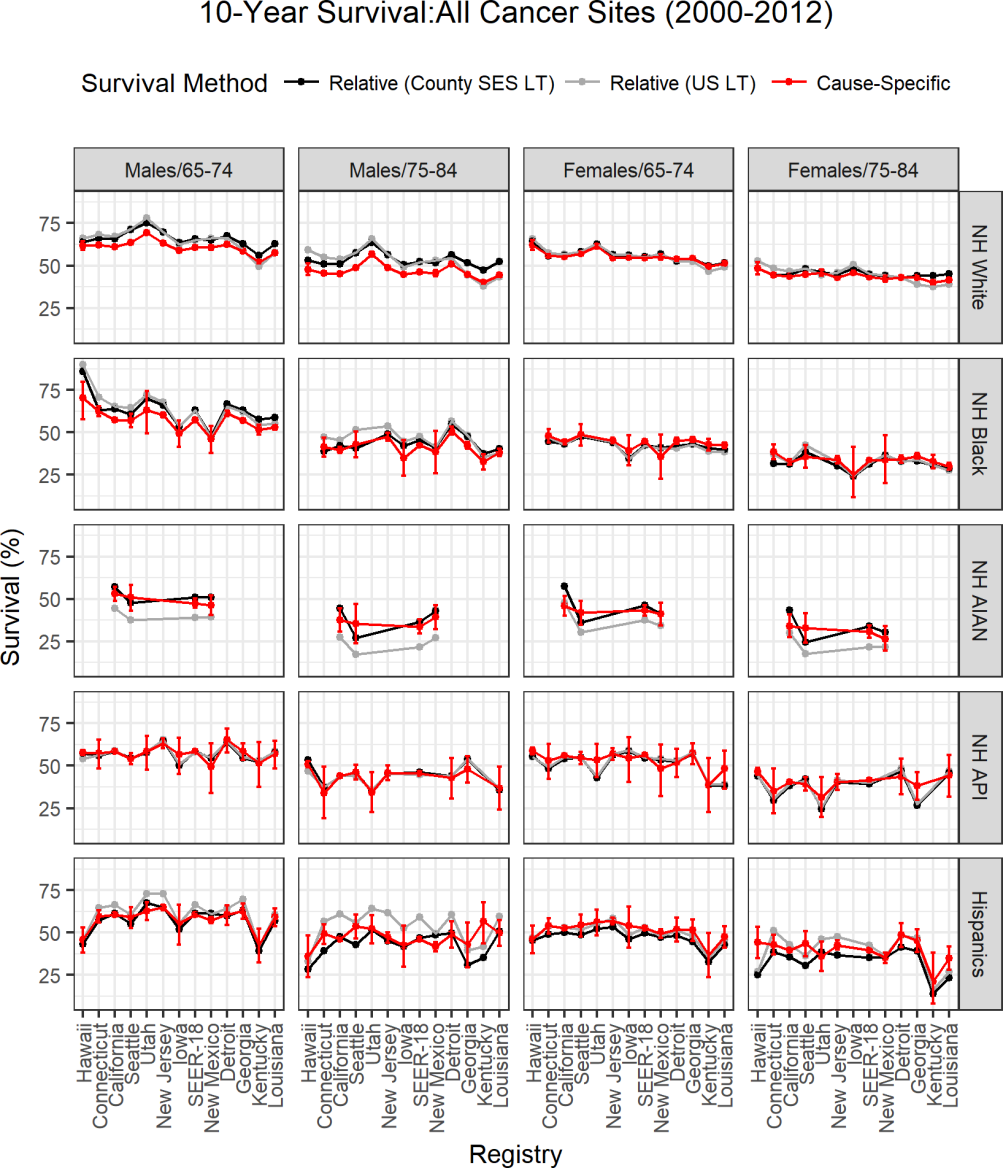
10-year relative survival using the County SES-LT (black) and the US-LT (gray), and 10-year cause-specific survival and its respective 95% confidence intervals (red) for patients diagnosed with any cancer between 2000 and 2012 by sex, age, and race. Registries are grouped from the highest SES (left of SEER-18) to the lowest SES (right of SEER-18).

Fig 5 displays 10-year relative survival, using County SES-LT and US-LT and cause-specific survival for all cancer sites combined by sex, age and race. We chose to report 10-year survival as differences are maximized. Overall differences between the three survival measures were small. We highlight the main systematic differences. Among whites, especially men aged 75–84, the US-LT overestimates and underestimates relative survival compared to County SES-LT in high and low SES areas, respectively. Both County SES-LT relative survival and cause-specific survival show less of a gradient compared to US-LT relative survival, underscoring the fact that national average LT may increase differences in relative survival. Like expected survival, the largest differences between County SES-LT and US-LT relative survival were observed for AIAN and Hispanics cancer patients. For the AIAN cancer patients the County SES-LT relative survival is much higher, often higher than 10%, points, compared to relative survival from the US-LT and closer to cause-specific survival. For Hispanics, County SES-LT relative survival was lower relative to the US-LT relative survival especially for cancer patients aged 75–84 and closer to cause-specific survival.

Table 2 presents a summary of the comparisons between 10-year relative survival using US-LT, County SES-LT and 10-year cause specific survival for cancer patients diagnosed in the aggregated SEER-18 areas between ages 75–84 years by race. Except for whites, 10-year County SES-LT relative survival was closer to 10-year cause-specific survival. The largest differences were seen among AIAN and Hispanics. Relative survival using County SES-LT was 7.3% and 6.7% survival points closer to cause-specific survival compared to US-LT relative survival for AIAN and Hispanics cancer patients, respectively.

**Table 2:** 10-year relative using US-LT and County SES-LT compared to 10-year cause specific survival for cancer patients diagnosed in the SEER-18 areas in 2000–2012 between ages 75 and 84 years both sexes combined.

	10-year survival (%)			Absolute difference	
Race	Relative US LT (a)	Relative SES LT (b)	Cause-Specific (c)	|a-c|	|b-c|
NH Whites	48.5	48.7	44.8	3.7	3.9
NH Blacks	39.5	38.2	37.9	1.6	0.3
NH AI/AN	21.8	35.2	32.2	10.3	3.0
NH API	42.6	43.1	43.7	1.1	0.6
Hispanics	51.3	41.4	43.0	8.3	1.6

NH = Non-Hispanics.

## Discussion

In this study, we developed an extensive set of life tables (LT) representing mortality patterns in the United States over three decades by race, ethnicity, sex, geography, and county-level SES. These tables can help calculate life expectancy and improve relative survival estimates. Despite gains in life expectancy between 1992 and 2012, this study shows that large disparities in life expectancy continue to exist among sex, race, state, and socio-economic (SES) groups. Asian or Pacific Islanders (API) had the highest life expectancy, followed by Hispanics, whites, blacks, and American Indian and Alaska Natives (AIAN). Black and AIAN men had the lowest life expectancy [25]. Between 1992 and 2012, the largest increases in life expectancy were observed among black men [26], and no increase was observed for AIAN women. Our findings suggest that differences in race, geography, and SES have a greater effect on life expectancy and on County SES-LT relative survival among males compared to females. Previous research has shown that most variation in life expectancy is due to differences in health behaviors, including smoking and obesity [12]. Thus the larger impact of race, geography, and SES on males life expectancy may be attributed to the fact that males have a higher and larger geographical variability in smoking prevalence compared to females [27].

The main use of these life tables is for the reporting of relative survival from U.S. cancer registries. Differences between relative survival calculated using the County SES-LT versus the US-LT were in general small, particularly for younger cancer patients, for areas with SES comparable to the national average (e.g. SEER-18), and for survival of 5-years or less (data not shown). Differences were larger for older ages, whites in high or low SES areas, AIANs, Hispanics, APIs in Hawaii, and long term survival. Compared to US-LT, the new County SES-LT provide lower expected survival in lower SES areas and higher expected survival in higher SES areas. Consequently, relative survival using the County SES-LT is lower in areas with high SES and higher in areas with low SES, decreasing differences in relative survival. This was more evident for whites and for males, as LT were more detailed and included both state and the full 5 levels of SES at the county level. Specific AIAN LT improved and increased estimates of AIAN relative survival by more than 10% survival points in most cases. The County SES-LT relative survival estimates were in general closer to cause-specific survival than US-LT relative survival, demonstrating that County SES-LT better captured background mortality especially in high versus low SES areas, male patients diagnosed at older ages, and among AIAN and Hispanics.

Comparisons between relative survival and cause-specific survival are challenging[15, 28], since both can be subject to bias. We used all cancer sites and included only cancer patients with one tumor to improve comparability between relative versus cause-specific survival. This comparison also shows that although the County SES-LT were closer to cause-specific survival, there were still some systematic differences. For example, for white males aged 75–84, the County SES-LT relative survival was parallel but higher than cause-specific survival by an average 7% points. The large percent of prostate cancers (30%) diagnosed among men, compared to all other cancers, may explain this difference along with the healthy screening effect. The healthy screening effect postulates that people detected with cancer through screening may have a higher life expectancy than the general US population, perhaps because of better overall health, greater access to health care, or healthier lifestyles. The healthy screening effect was most recently demonstrated among prostate cancer patients in the Prostate, Lung, Colorectal and Ovarian (PLCO) Cancer Screening Trial. Participants in this trial had a 30–50% lower mortality rate for heart disease, injury, and kidney disease than expected in the general population[29].

There were limitations to our study. We used mortality data linked to an SES indicator at the county level, which is subject to large variability in population size. For large counties, such as Los Angeles County, California, with a population close to 10 million, the SES index represents the average of SES in the county for all residents and does not have the required specificity to characterize the diversity of SES in the county. Analysis at the census tract level would be more attractive, since census tract populations are more homogeneous, however county is the smallest geographical level for which U.S. mortality data are available. The composite SES index summarizes a full range of SES characteristics to simplify the modeling but still accounts for variation in SES level with geography and race. The quintiles derived are relative measures of SES in the U.S., and useful for comparing inequalities between counties. The quintiles also showed a consistent pattern, in which higher life expectancies were associated with higher SES quintiles and vice versa.

Importantly, modeling separately for each race at the individual level already considers important SES differences. For example, DC represented by one county (Q4), provided the highest life expectancy for white men (82 years) and the lowest for black men (66 years). Second, because of a small number of deaths, we varied the models with respect to geography and SES levels by race groups and restricted analyses to ages 30 to 84 to provide robust LT estimates. Although this approach may not have captured all the variability in the mortality in the older and younger age groups, restricting the modeling to ages 30 to 84 and borrowing information from the national NCHS LT provided more stable and less biased estimates of LT in those age groups. A previous study has shown that state life tables using mortality data beyond ages 85 provided unreliable estimates of relative survival [11]. The NCHS national life tables are more reliable for older age groups because they use Medicare data, not available to us, to provide a more accurate determination of age of death for older individuals. Because mortality at younger ages is very low, the impact of life tables on relative survival from cancer patients diagnosed at younger ages is very small. Our estimates based on national LT are more robust and not subject to potential biases due to data variability at the younger ages when mortality is small.

We also restricted estimation of the AIAN LT to CHSDA areas, similar to other studies [17]. Life expectancy for the AIAN population in CHSDA areas, which are predominantly rural, isolated areas with limited access to employment and health care, may not well represent the total AIAN populations. However, estimates not restricting to CHSDA areas would result in unrealistically high estimates of AIAN life expectancy. Our estimates are similar to Arias et al.[17], a life expectancy of 68.3 in 2010 versus a life expectancy of 68 years in 2007–2009 for non-Hispanic AIAN in Arias et al. [17].

The County SES-LT included calendar year, age, sex, race, area of residency and County SES when possible. We were not able to include other variables at the county level, such as risk factors that may affect other causes of death, e.g. smoking or obesity, or variables related to access to health care, as these data are not available for the full range of years and for all counties. Previous studies have shown that LT adjusting for higher, smoking-related background mortality, had little or modest impact on relative survival estimates for lung cancer [30, 31].

Strengths of our study include the large sample size, the population-based setting, and the fact that the LT are an extensive representation of the varying mortality patterns in the U.S. over three decades by race, ethnicity, sex, geography and county-level SES. Our study highlighted the importance of LT by geography and other factors for comparisons and calculation of relative survival among different cancer registries, due to the disparities seen in life expectancy across different subgroups in the U.S. Analyses of life expectancies from other studies provided comparable results[12, 25–26], giving validity to these LTs. The comparisons of relative and cause-specific survival showed that the County SES relative survival were closer to cause-specific survival and had a smaller gradient between low versus high SES areas, reducing differences in relative survival. This substantiated the fact that relative survival using a national average background mortality (thus same denominator) overestimates and underestimates survival in high and low SES areas, respectively.

In summary, we have shown that differences between relative survival using the SES and the US-LT were in general small. However, relative survival using County SES-LT were closer to cause-specific survival and improved estimates for some demographic groups, in particular Hispanics, AIAN, populations in higher or lower SES areas/states, and among older male cancer patients. Investigations using SEER data to examine time trends in cancer survival and disparities in cancer survival by race, ethnicity, and SES are common. Studies of cancer survival using life tables that do not properly account for differences in background mortality by these factors may mischaracterize trends and overstate the magnitude of disparities. Recently, the North American Association of Central Cancer Registries (NAACCR) began to publish cancer survival estimates on a larger number of U.S. state registries in the Cancer in North America annual reports [13]. We suggest using the life tables described in this paper as default for cancer relative survival using U.S. data, including the CDC’s National Program of Cancer Registries, the SEER registries, and by researchers conducting international studies that include U.S. data [14].The use of these life tables will advance population-based cancer surveillance and research by contributing standardized and more accurate relative survival estimates.

## Supporting information

S1 Table Life table model description for each state and race. (XLSX)
